# Angiotensin II type 2 receptor signaling significantly attenuates growth of murine pancreatic carcinoma grafts in syngeneic mice

**DOI:** 10.1186/1471-2407-10-67

**Published:** 2010-02-24

**Authors:** Chiyo Doi, Noboru Egashira, Atsushi Kawabata, Dharmendra Kumar Maurya, Naomi Ohta, Deepthi Uppalapati, Rie Ayuzawa, Lara Pickel, Yuka Isayama, Deryl Troyer, Susumu Takekoshi, Masaaki Tamura

**Affiliations:** 1 Department of Anatomy & Physiology, Kansas State University, College of Veterinary Medicine, Manhattan, KS 66506, USA; 2 Department of Pathology, Tokai University School of Medicine, Isehara, Kanagawa 259-1193, Japan

## Abstract

**Background:**

Pancreatic cancer is one of the most aggressive human malignancies, with a very poor prognosis. To evaluate the effect of angiotensin II (Ang II) type 2 receptor (AT_2_) expression in the host's body on the growth of pancreatic carcinoma, we have investigated the growth of mouse pancreatic ductal carcinoma grafts in syngeneic wild type and AT_2 _receptor-deficient (AT_2_-KO) mice.

**Methods:**

The role of AT_2 _receptor-signaling in stromal cells on the growth of murine pancreatic carcinoma cells (PAN02) was studied using various *in vitro *and *in vivo *assays. *In vivo *cell proliferation, apoptosis, and vasculature in tumors were monitored by Ki-67 immunostaining, TUNEL assay, and von Willebrand factor immunostaining, respectively. In the co-culture study, cell proliferation was measured by MTT cell viability assay. All the data were analyzed using t-test and data were treated as significant when *p *< 0.05.

**Results:**

Our results show that the growth of subcutaneously transplanted syngeneic xenografts of PAN02 cells, mouse pancreatic ductal carcinoma cells derived from the C57/BL6 strain, was significantly faster in AT_2_-KO mice compared to control wild type mice. Immunohistochemical analysis of tumor tissue revealed significantly more Ki-67 positive cells in xenografts grown in AT_2_-KO mice than in wild type mice. The index of apoptosis is slightly higher in wild type mice than in AT_2_-KO mice as evaluated by TUNEL assay. Tumor vasculature number was significantly higher in AT_2_-KO mice than in wild type mice. *In vitro *co-culture studies revealed that the growth of PAN02 cells was significantly decreased when grown with AT_2 _receptor gene transfected wild type and AT_2_-KO mouse-derived fibroblasts. Faster tumor growth in AT_2_-KO mice may be associated with higher VEGF production in stromal cells.

**Conclusions:**

These results suggest that Ang II regulates the growth of pancreatic carcinoma cells through modulating functions of host stromal cells; Moreover, Ang II AT_2 _receptor signaling is a negative regulator in the growth of pancreatic carcinoma cells. These findings indicate that the AT_2 _receptor in stromal fibroblasts is a potentially important target for chemotherapy for pancreatic cancer.

## Background

Pancreatic cancer is one of the leading causes of cancer death in many countries, including the United States. Pancreatic ductal adenocarcinoma (PDAC) constitutes approximately 90% of all primary malignant tumors arising from the pancreatic gland. Of all gastrointestinal malignancies, pancreatic adenocarcinoma is the second most common cause of death from cancer [1-3]. Pancreatic cancer is an aggressive malignant cancer with a high metastatic rate and is an almost uniformly lethal disease in humans [3-5]. Of affected patients, 60% have liver metastasis, malignant ascites, or other evidence of tumor spread at the time of diagnosis [6]. The 5-year survival rate in the United States is less than 5% [3].

The renin-angiotensin system is one of the phylogenetic hormone systems and plays a key role in the regulation of cardiovascular homeostasis, which maintains arterial blood pressure and fluid and electrolyte homeostasis [7,8]. Angiotensin II (Ang II), an octapeptide hormone, is the key effector in the renin-angiotensin system. Ang II has two well-defined receptors: Ang II type 1 (AT_1_) and type 2 (AT_2_) receptor [9]. The AT_1 _receptor is widely expressed in a variety of adult tissues. AT_1 _receptor-mediated signaling is responsible for most Ang II-dependent actions in cardiovascular and renal tissues. Responses of the AT_1 _receptor are typically associated with stimulation of growth factor receptors leading to cell growth, proliferation, cell migration, apoptosis, and gene expression [10,11]. These effects are executed through a heterotrimeric G protein-coupled receptor, which mediates Ang II transactivated epidermal growth factor (EGF)-induced activation of MEK (MAPK kinase 1) and ERK [12]. The AT_2 _receptor, the second major isoform of the Ang II receptor, is primarily expressed in the mesenchyme of the fetus and to a limited extent in adult tissues [13]. It is, however, inducible and functional under pathophysiologic conditions [14-17]. The AT_2 _receptor mediates signals that counteract the AT_1 _receptor-mediated biological actions [18-20]. In addition, the AT_2 _receptor is known to inhibit cell proliferation and stimulate apoptosis in cardiovascular and neuronal tissues *in vitro *[21]. However, the relationship between the AT_2 _receptor and cancer has yet to be clarified. Our previous studies revealed that chemical carcinogen-induced tumorigenesis in mouse colon [22] and lung [15] was significantly attenuated by AT_2 _receptor deficiency. Since AT_2 _receptor expression has been noted in various stromal fibroblasts [23,24] and is inducible in the pancreas in pathological conditions [25], AT_2 _receptor deficiency may also influence pancreatic cancer growth. In addition, Ang II receptor antagonists and angiotensin I-converting enzyme inhibitors currently used for human clinical hypertension treatment attenuate growth of human cancer cells in experimental animals [26-30] and may reduce the risk of several human cancers[31]. This suggests that AT_2 _receptor expression potentially plays an important role in cancer.

In the present study, we subcutaneously inoculated pancreatic ductal carcinoma cells in syngeneic AT_2_-KO and wild type mice and examined tumor growth, cell proliferation, and apoptosis. In addition to the *in vivo *study, we also studied the effect of stromal fibroblasts, which were prepared from either AT_2_-KO or control wild type mice, on PAN02 cancer cell growth *in vitro*. These studies revealed that Ang II AT_2 _receptor signaling in stromal cells plays an important regulatory role in the growth of pancreatic carcinoma cells.

## Methods

### Materials

Ang II was purchased from Peninsula Laboratories Inc. (San Carlos, CA). The AT_1 _receptor blocker Losartan was a gift from Dr. Tadashi Inagami (Vanderbilt University Medical Center); the AT_2 _receptor blocker PD123319 was purchased from Sigma Chemical Co. (St. Louis, MO). Rabbit anti-human von Willebrand factor (vWF) and rat anti-mouse Ki-67 antibodies were purchased from DakoCytomation (Glostrup, Denmark). Rabbit anti-human vascular endothelial cell growth factor (VEGF) and rabbit anti-human GAPDH antibodies were from Santa Cruz Biotechnology, Inc. (Santa Cruz, CA). A biotin-conjugated secondary antibody was purchased from Jackson ImmunoResearch (West Grove, PA). Avidin-biotin peroxidase complex (ABC) reagents was from Vector Laboratories (Burlingame, CA). ApopTag^® ^Plus Peroxidase *In Situ *Apoptosis Detection Kit was from Chemicon International, Inc. (Tokyo, Japan). Bio-αRat biotin-conjugated secondary antibody was from Jackson ImmunoResearch, (West Grove, PA). A horseradish peroxidase-conjugated anti-rabbit IgG secondary antibody was from Amersham Biosciences (Piscataway, NJ). All other chemicals were of analytical grade.

### Cell culture

The PAN02 murine pancreatic adenocarcinoma cell line was obtained from the National Cancer Institute and maintained in RPMI-1640 medium supplemented with 10% fetal bovine serum (FBS), 2 mM L-glutamine, 100 U/ml penicillin, and 100 μg/ml streptomycin.

Primary cultured mouse skin fibroblasts (MSFs) from wild type and AT_2_-KO mice were prepared from 24 to 48 hour old C57BL/6J mouse pups following an established method [14]. MSFs were cultured in DMEM/Ham's F-12 medium (1:1) supplemented with 10% FBS, 100 U/ml penicillin, and 100 μg/ml streptomycin. All cells were incubated in 5% CO_2 _humidified air at 37°C.

### Animals and genotyping

Hemizygous AT_2_-KO mutant (Agtr2-/y) mice were generated as described previously [15]. These mice were backcrossed with wild type C57BL/6J (The Jackson Laboratory, Bar Harbor, MA) for 17 generations such that the genetic background of the mice is susceptible to our pancreatic cancer syngeneic model. Wild type littermates served as controls. Genotypes were confirmed by the PCR method using extracted tail DNA. Briefly, published sequences [19,32] were used to synthesize primers for the AT_2 _receptor (forward 5'-CACCAGCAGAAACATTAC-3' and reverse 5'-AACACAGCTGTTGAATCC-3') and the neomycin resistance (Neo-r) gene product (forward 5'-AGCCAACGCTATGTCCTGAT-3' and reverse 5'-AGACAATCGGCTGCTCTGAT-3'). Extracted tail DNA (10-20 ng) was amplified (35 cycles) at 95°C for 1 minute (denaturation), at 58°C for 1 minute (annealing), and at 72°C for 1 minute (elongation) with 0.5 nmol/L of each primer, 1.25 units DNA polymerase, and 0.2 mmol/L deoxynucleotide triphosphates in PCR buffer. PCR products of the AT_2 _receptor (478 bp) and Neo-r gene product (593 bp) were visualized by 1% agarose gel electrophoresis. AT_2 _(+) and Neo-r (-), AT_2 _(+) and Neo-r (+), and AT_2 _(-) and Neo-r (+) were assigned as wild type, heterozygote, and AT_2_-KO, respectively. All animals were maintained in a humidity- and temperature-controlled room on 12-hour light/dark cycles. All procedures for handling animals were approved by the Institutional Committee for Animal Care and Use of Kansas State University.

### Pancreatic cancer syngeneic model

Seven to nine week-old AT_2_-KO/C57BL/6J mice and wild-type littermates were anesthetized with isoflurane. Cells were trypsinized and washed with PBS. Five million cells in 200 μl PBS were subcutaneously inoculated into each flank using a 1 ml syringe with a 27G needle [16]. The tumor size was measured by caliper every three days and the volume was calculated using the formula (short diameter)^2 ^× (long diameter) × 0.5 [17].

At the end of the experiments, the mice were sacrificed by cervical dislocation under anesthesia. The tumors were dissected and weighed. For histological assessment, the specimens were fixed in 10% formalin, embedded in paraffin, and sectioned for histopathological analysis.

### Immunohistochemical analysis

Tissue sections of 4 μm thickness were prepared for all staining. Slides were dewaxed and rehydrated before staining. The heat-induced antigen unmasking was performed in Citra Plus Solution, pH 6.0 (BioGenex, San Ramon, CA) for 5-10 minutes using an autoclave oven. Sections were then incubated with 0.3% hydrogen peroxide in methanol for 20 minutes to block endogenous peroxide activity. The dilution of antibodies for Ki-67, von Willebrand factor (vWF) and VEGF was 1:50, 1:100, and 1:50, respectively. Sections were incubated with the primary antibodies for 60 minutes at room temperature. In immunostaining for Ki-67, sections were incubated with biotin-conjugated secondary antibody (Jackson ImmunoResearch, West Grove, PA) followed by reaction with the avidin-biotin peroxidase complex (ABC) reagent (Vector Laboratories, Burlingame, CA) for 30 minutes at room temperature. In immunostaining for vWF, an ABC kit (Vector Laboratories) was used. Peroxidase activity was visualized with 3,3'-diaminobenzodine tetrahydrochloride (Sigma Chemical Co). Sections were lightly counterstained with Hematoxylin solution (Merck KGaA, Darmstadt, Germany).

### TUNEL assay

To determine cell death, apoptotic cells in paraffin sections were detected by TUNEL (Terminal Deoxynucleotidyltransferase-Mediated dUTP Nick End Labeling) assay using the Apop Taq Plus Peroxidase *In Situ *Apoptosis Detection Kit (Millipore Corporation, Billerica, MA) according to the manufacturer's instructions. Sections were counterstained with Methyl green solution (Nacalai Tesque, Inc., Kyoto, Japan).

### Image analysis

Ki-67 or TUNEL positive cell numbers and whole cell numbers (as background) in five randomly selected fields were counted by two independent observers. The VEGF positive cell area in five randomly selected fields was evaluated using NIH digital-image analyzing software, Image J 1.37v, (NIH, Bethesda, MD).

### Evaluation of the effect of angiotensin II and fibroblasts on the growth of PAN02 cells

Primary cultured MSFs (100 cells/well, 96-well plate) from wild type or AT_2_-KO mice were incubated in serum-free medium in 5% CO_2 _humidified air at 37°C. Following 24 hours incubation, PAN02 cells (400 cells/well) were added to the culture plate and co-cultured with the wild-type or AT_2_-KO MSFs in DMEM/Ham's F12 medium (1:1) containing 10% FBS. One day after co-culture, the cells were treated with Ang II (10 nM) for 48 hours in the presence of the AT_2 _receptor-specific antagonist PD123319 (10 μM). The degree of cell proliferation was evaluated by MTT assay. In brief, 10 μl MTT solution (5 mg/ml) was added to each well 4 hours prior the end of the incubation. Formazan crystals formed in the cells were dissolved by adding 100 μl of MTT solvent (0.01 N HCl in 10% SDS). The absorbance was measured at 550 nm by spectrometer 24 hours after incubation at 37°C with the MTT solvent.

### Evaluation of the effect of AT_2 _receptor over-expression in fibroblasts on co-cultured PAN02 cell growth

MSFs from wild type or AT_2_-KO mice were seeded in T25 flasks. After cell attachment, the medium was changed to serum-free DMEM. After three hours in the serum-free medium, the medium was changed to 875 μl DMEM containing 5% FBS and either adenoviral AT_2 _receptor (Ad-AT_2_, 25 MOI) or adenoviral Lac Z (Ad-Lac Z, 25 MOI). The cells were incubated in 5% CO_2 _at 37°C; the flasks were rocked every 15 minutes for 3 hours. After incubation with the vectors, DMEM/Ham's F12 (1:1) containing 10% FBS was added and the cells were further incubated for an additional 24 hours at 37°C in 5% CO_2_. Both untransfected and transfected MSFs were co-cultured with PAN02 cells (400 cells/well) as described above. The extent of cell proliferation was evaluated by MTT assay.

### Gene expression analysis using real-time PCR

Total RNA was extracted from cells using TRIzol reagent (Invitrogen). Genomic and complementary DNA was removed using RQ1 RNase-free DNase (Promega, Madison, WI) according to the manufacturer's instructions. Real-time PCR was carried out using an iScript One-Step RT-PCR Kit with SYBR Green (Bio-Rad, Hercules, CA), and the reactions were conducted on the real-time PCR detection system iCycler (Bio-Rad). The results were quantified as C_t _values, where C_t _is defined as the threshold cycle of PCR at which the amplified product is first detected and signifies relative gene expression (the ratio of target/control). The AT_2 _primers were 5'-AGC CAA GGC CAG ATT GAA GA-3' (forward) and 5'-GCC ACC AGC AGA AAC ATT ACC-3' (reverse), the AT1 primers were 5'-GGC AGC ATC GGA CTA AAT GG-3' (forward) and 5'-CCA GCT CCT GAC TTG TCC TTG-3' (reverse), and the 18S ribosome RNA primers were 5'-TCG CTC CAC CAA CTA AGA AC-3' (forward) and 5'-GAG GTT CGA AGA CGA TCA GA-3' (reverse).

### Western blot analysis

Total cellular protein was prepared according to our routinely used protocol [33]. The membrane was incubated with the antibody against VEGF at a 1:250 dilution in TBST with 0.1% nonfat dry milk for 1 hr at room temperature. Then, the membrane was incubated with a horseradish peroxidase-conjugated anti-rabbit IgG secondary antibody at a 1:2000 dilution in TBST with 0.1% nonfat dry milk for 1 hr at room temperature. The protein expression signal was detected with Pierce SuperSignal Western Blotting substrate. GAPDH was used as the loading control of sample by reprobing with an anti-GAPDH antibody at a 1:12000 dilution.

### Statistical analysis

Results are expressed as mean ± standard error of the mean (SEM). For statistical analysis, a Microsoft Excel Data Analysis tool, t-test, was used. The critical value was 95%, and significance was defined as *p *< 0.05.

## Results

### Growth of mouse pancreatic ductal adenocarcinoma grafts was faster in syngeneic AT_2_-KO mice than in wild type mice

**Figure 1:**
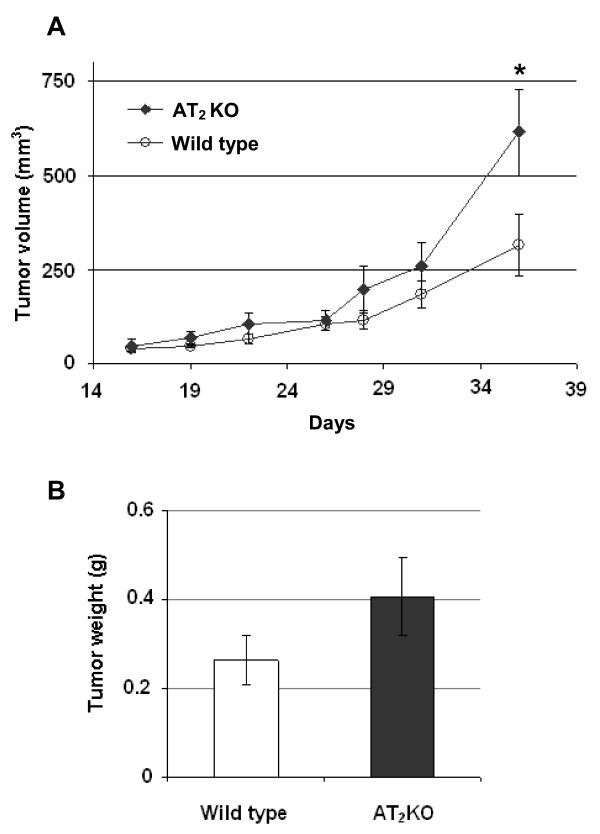
**The effect of AT_2 _receptor deficiency on the growth of murine pancreatic ductal carcinoma (PAN02) xenografts was evaluated in syngeneic mouse model**. PAN02 cells (5 × 10^6 ^cells suspension in 200 μl PBS) were inoculated into both flanks in either AT_2_-KO (n = 6) or wild type mice (n = 6). Tumor volume was calculated after measuring tumor diameter every three to four days using a caliper (A). Tumors were carefully removed at the end of the study and the tumor weight was determined (B). Data are presented as means ± SEM of twelve tumors from six mice. * P ≤ 0.05 as compared to the level of wild type mouse group.

To investigate the influence of the AT_2 _receptor on tumor growth, we inoculated PAN02 cells into both flanks of syngeneic AT_2_-KO (n = 6) and wild-type (n = 6) C57BL/6 mice. Our results showed that tumor growth was significantly faster in AT_2_-KO mice than in the control wild type mice (Figure 1). At the time of sacrifice, AT_2_-KO mice had significantly larger tumors than wild type mice (Figure 1), with a mean tumor volume of 642.73 and 263.37 mm^3^, respectively (P < 0.05). Since real time PCR revealed that primary cultured wild type mouse skin fibroblasts express the AT_2 _receptor, but PAN02 cells do not (Table 1), these results indicate that the host stromal AT_2 _receptor is involved in the growth of PAN02 xenografts.

**Table 1:** Expression of both angiotensin II AT_1 _and AT_2 _receptors was detected in wild-type mouse skin fibroblasts (MSF) but not in PAN02 cells

Genes	AT_1_		AT_2_		18S	
			
Serum	+	-	+	-	+	-
MSF	22.8	23.5	28.1	20.6	11.9	12.8
PAN02	29.6	29.7	29.4	29.7	10.0	11.3
H_2_0	30.6		30.0		29.8	

Ang II receptor expression in wild-type MSF and PAN02 cells was determined by real-time PCR and expressed as threshold cycle (Ct) value. The 18S ribosomal RNA was used as an internal standard. The Ct value indicates the fractional cycle number at which the amount of amplified target reaches a fixed threshold. Cells were cultured in either serum-containing or serum-free medium for two days, total RNA was extracted, and the receptor expression was determined. The values represent the average of triplicate determinations. Serum-free culture is known to increase AT_2 _expression in cultured cells [34].

### The cell proliferation index was significantly higher in AT_2_-KO mouse tumors than in wild type mouse tumors

**Figure 2:**
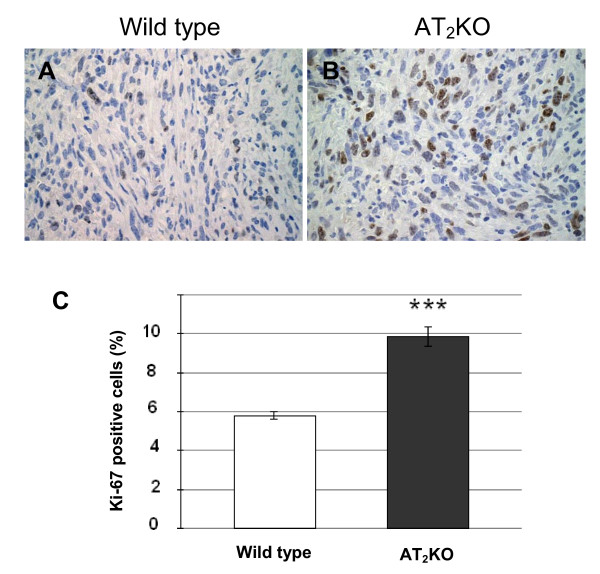
**Cell proliferation in tumor tissues was analyzed by counting anti-Ki-67 antibody positive cells in wild type (A) and in AT_2_-KO mice (B)**. Quantitative analysis of cell proliferation (C) was carried out by counting anti-Ki-67 antibody positive and negative cells in five random view fields; mean % positive cells was calculated from this raw data for graphical presentation. Data are presented as percentages ± SEM of Ki-67 positive cells/field. Original magnification of each panel is 400 ×. * P ≤ 0.001 as compared to the level of wild type mouse group.

To evaluate cell proliferation in tumors in both types of mice, the cell growth index was analyzed using an anti-Ki-67 antibody. More Ki-67 positive cells were detected in AT_2_-KO mouse tumor sections than in wild type mouse tumor sections (Figure 2). A detailed examination of the morphology of the Ki-67 positive cells revealed that these cells are tumor cells. In quantitative analysis, the percentage of Ki-67 positive cells is significantly higher in AT_2_-KO mouse tumors than in wild type mouse tumors (P ≤ 0.001, Figure 2C). This result indicates that PAN02 tumor growth is faster in AT_2_-KO mice than in wild type mice.

### The apoptotic index was lower in AT_2_-KO mouse tumors than in wild type mouse tumors

**Figure 3:**
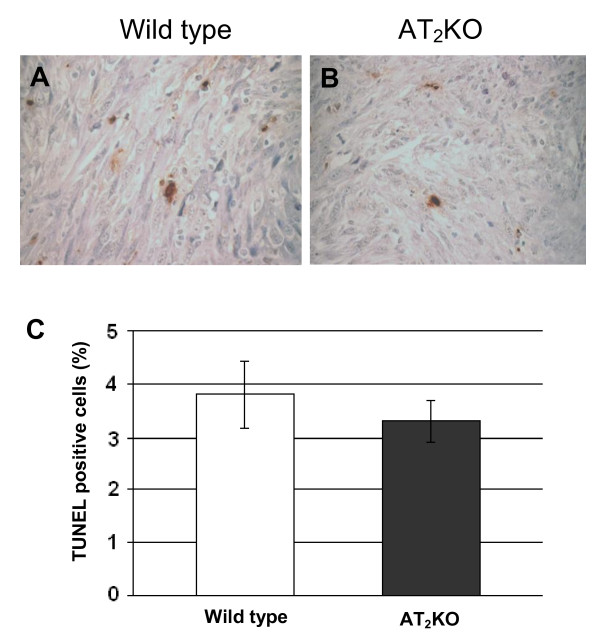
**Apoptosis in tumor tissues was analyzed by visualizing TUNEL positive cells in wild type (A) and AT_2_-KO (B) mouse tumors**. Quantitative analysis of apoptosis (C) was carried out by counting TUNEL positive and negative cells in five random view fields; mean % positive cells was calculated from this raw data for graphical presentation. Data are presented as percentages ± SEM of TUNEL positive cells/field. The original magnification of each panel is 400 ×.

The *in vivo *apoptotic index in tumor tissue from AT_2_-KO and wild type mice was examined by a Terminal Deoxynucleotidyltransferase-Mediated dUTP Nick End Labeling (TUNEL) assay. As shown in Figure 3, although slightly more TUNEL positive cells were detected in tumors from wild type mice than in tumors from AT_2_-KO mice, the difference between the two values was statistically not significant (P = 0.21). In addition, apoptotic cells appeared to be a mixture of tumor cells and tumor-infiltrating leukocytes. This result suggests that apoptosis may not be a major contributor to the different tumor growth in the two groups.

### Histochemical analysis indicated higher vascular density in AT_2_-KO mouse tumors than in wild type mouse tumors

**Figure 4:**
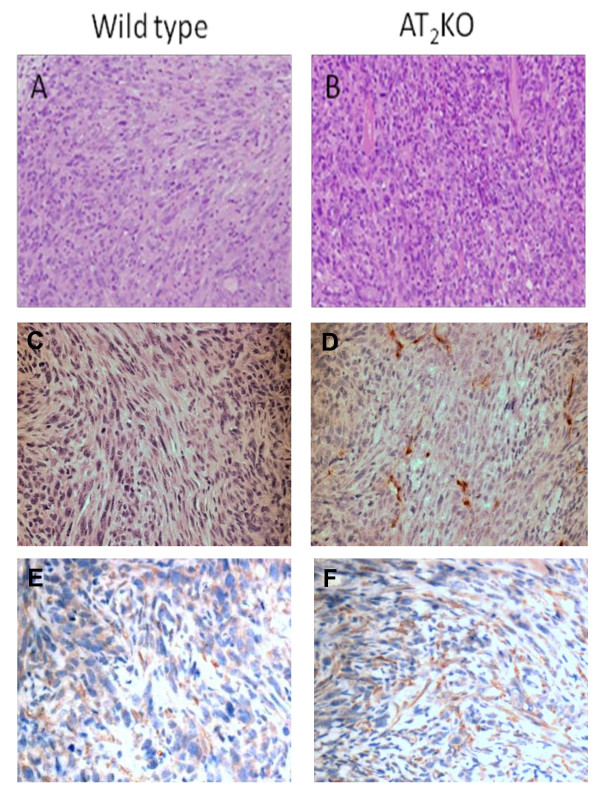
**Tumor morphology in wild type (A) and AT_2_-KO mice (B) was studied after hematoxylin and eosin staining**. Tumor vessel density in wild-type (C) and AT_2_-KO mouse tumors (D) was studied after endothelial cell staining using vascular endothelial cell-specific anti-von Willebrand factor antibodies. Average tumor vasculature numbers in AT2-KO mouse tumors (8.3 ± 0.1/field) were significantly higher than in wild type tumors (2.1 ± 0.5/field, p < 0.05). VEGF expression in wild-type (E) and AT_2_-KO mouse tumors (F) was also studied after VEGF immunostaining using anti-VEGF antibodies. The original magnification of panels A and B is 200 × and panels C, D, E, and F is 400 ×.

Overall histochemical analysis of the tumors indicates that they are undifferentiated carcinoma. Occasionally, sarcoma-like morphology was observed in the tumor tissue (Figures 4A and 4B). Tumors in both mouse types contain very little stroma. However, vascular endothelial cell staining by anti-von Willebrand factor antibodies revealed that tumors in the AT_2_-KO mice contain significantly more microvasculatures than tumors in wild type mice (Figure 4C and 4D). Average tumor vasculature numbers in five randomly chosen fields in wild type and AT_2_-KO mouse tumors was 2.1 ± 0.5 and 8.3 ± 0.1/field, respectively (p < 0.05). Furthermore, immunostaining against VEGF revealed that the cells with morphology similar to fibroblasts in tumor stroma were the primary VEGF positive cells in the tumors. Although VEGF expression in tumor cells was visible, this expression was not as strong as in fibroblastic cells. VEGF positive cells were more abundant in AT_2_-KO mouse tumors than in wild type mouse tumors (4.2 vs. 7.8/field, respectively, P = 0.15), although the difference between two groups was not statistically significant due to large variation. These results suggest that faster tumor growth in AT_2_-KO mice may be associated with development of tumor microvasculature. Results further suggest that the tumor stromal fibroblasts may play an important role in tumor growth.

### Angiotensin II stimulated growth of PAN02 cells co-cultured with fibroblasts, and this stimulation was further increased by an AT_2 _receptor specific antagonist

**Figure 5:**
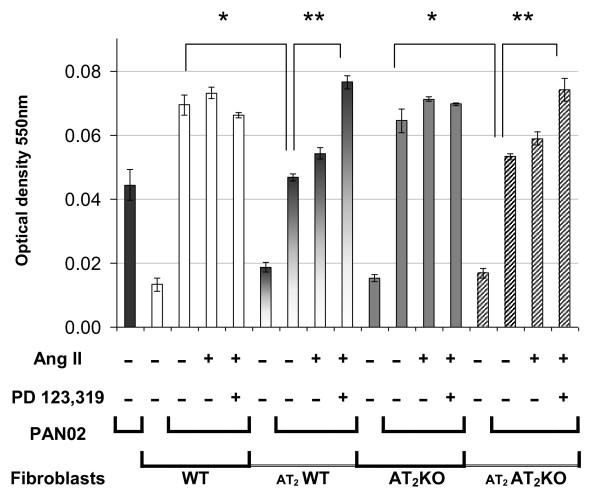
**The effect of Ang II and an AT_2 _receptor antagonist on cell proliferation, as determined by MTT assay, of PAN02 cells (black solid bars) co-cultured with MSFs prepared from either wild type (WT) mice (untransfected WT fibroblasts, WT, open bars; and AT_2 _over-expressing WT fibroblasts, _AT2_WT, half-shaded bars) or AT_2_-KO mice (untransfected AT_2_-KO fibroblasts, AT_2_KO, dark grey bars; and AT_2 _over-expressing AT_2_-KO fibroblasts _AT2_AT_2_KO, hashed bars)**. Untransfected or AT_2 _transfected primary cultured mouse skin fibroblasts were cultured one day prior to the initiation of PAN02 co-culture. The ratio of fibroblasts to PAN02 cells was 1:4. The cells were treated with Ang II (10 nM) in the presence or absence of the AT_2_-specific antagonist PD123,319 (10 μM) as indicated in the figure. Cell proliferation was determined 72 h after Ang II treatment by MTT assay as described in the Methods. The average (means ± SEM) of three separate experiments is displayed in the histogram. Cell proliferation of PAN02 co-cultured with AT_2 _over-expressing fibroblasts was significantly lower than that with untransfected fibroblasts regardless of the cell source (*, P ≤ 0.05), whereas PAN02 proliferation was significantly increased when cells were treated with the AT_2 _antagonist PD123,319 (** P ≤ 0.01).

To evaluate the effects of Ang II and the AT_2 _receptor signaling on the growth of PAN02 cells *in vitro*, the effect of a low concentration of Ang II (10 nM) was examined on the growth of PAN02 cells co-cultured with MSFs prepared from either wild type or AT_2_-KO mice or with AT_2 _receptor over-expressing MSFs prepared from either wild type or AT_2_-KO mice. Since AT_2 _receptor expression is known to be attenuated in culture [34], AT_2 _receptor expression should be assured by the receptor over-expression. As shown in Figure 5, growth of PAN02 was significantly attenuated when the AT_2 _receptor was over-expressed in co-cultured MSFs. Ang II only slightly increased the growth of PAN02 cells regardless of cell sources (wild type or AT_2_-KO mice) or AT2 expression in MSFs. However, Ang II significantly increased cell growth of PAN02 co-cultured with AT_2_-over-expressing MSFs when cells were treated with the AT_2 _receptor-specific antagonist PD123319 (10 μM). This AT_2 _receptor blockade effect was not observed when control Lac Z transfected MSFs were used in this experiment (data not shown). Ang II or PD123319 treatment did not show any significant effect on the growth of MSFs derived from either wild type or AT_2_-KO mice (data not shown). These results indicate that AT_2 _expression in co-cultured MSFs plays a negative role in cell proliferation of PAN02 cells and this effect can be reversed by the AT_2 _receptor blockade.

### Angiotensin II attenuated VEGF production in fibroblasts, and this attenuation was blocked by an AT_2 _receptor specific antagonist

**Figure 6:**
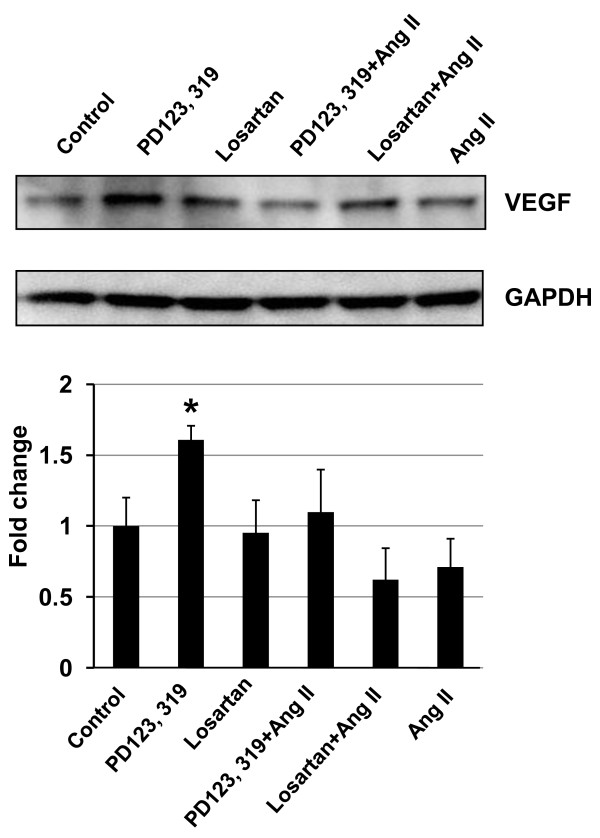
**The effect of Ang II and an AT_2 _receptor antagonist on VEGF protein expression in mouse fibroblasts was determined by Western blot analysis**. Primary cultured mouse skin fibroblasts were treated with Ang II (10 nM) in the presence or absence of the AT_1_- and AT_2_-specific antagonists Losartan (1 μM) and PD123,319 (10 μM), respectively, as indicated in the figure. VEGF protein was determined 48 h after Ang II treatment by Western blot analysis as described in the Methods. The average (means ± SEM) of two separate experiments is displayed in the histogram. Ang II attenuated VEGF expression (P = 0.1), but this attenuation was completely blocked by the AT_2 _antagonist PD123,319. However, PD123,319 treatment significantly increased VEGF production as compared to untreated control (*, P ≤ 0.05).

To evaluate a potential mechanism by which stromal cells regulate PAN02 tumor growth, the effect of a low concentration of Ang II (10 nM) on VEGF production in wild type MSFs was examined. As shown in Figure 6, Ang II attenuated VEGF protein expression in MSFs, and this attenuation was completely blocked when cells were pre-treated with the AT_2 _receptor-specific antagonist PD123319 (10 μM). PD123319 treatment alone slightly increased VEGF expression in MSFs (Figure 6). These results suggest that AT_2_-mediated Ang II signaling plays a negative role in VEGF expression in MSFs. This may imply that Ang II-dependent regulation of VEGF production in stromal cells may play an important role in PAN02 tumor growth.

## Discussion

Increasing evidence suggests that Ang II signaling plays an important role in carcinogenesis [15,19,22,35-37]. While AT_1 _receptor over-expression has been implicated in many types of cancers including pancreatic cáncer [11,12,38,39], the specific role of the AT_2 _receptor in carcinogenesis has not been rigorously elucidated. We have previously demonstrated the pro-oncogenic role of the AT_2 _receptor in carcinogen-induced colon and lung tumorigenesis in the mouse. In these models, the AT_2 _receptor appears to enhance carcinogen metabolism and increase tumorigenesis. However, the effect of AT_2 _receptor-mediated signaling on tumor growth is unknown. Since Ang II has been shown to stimulate tumor growth through the AT_1 _receptor [35,39,40], and since the AT_2 _receptor antagonizes the AT_1 _receptor [41,42], it is relevant to study the role of the AT_2 _receptor in tumor growth. Therefore, in this study we sought to evaluate the role of AT_2 _receptor expression in stroma in the growth of pancreatic ductal adenocarcinoma, the most common form of pancreatic cancer.

In the first study, we have examined the growth of PAN02 adenocarcinoma cells in AT_2_-KO and wild type mice and found that the growth of PAN02 xenografts is significantly faster in AT_2_-KO mice than in wild type mice (Figure 1). The degree of cell proliferation and the index of apoptosis were measured by anti-Ki-67 staining and TUNEL assay, respectively. It was found that anti-Ki-67 positive staining was significantly higher in AT_2_-KO mouse tumors than in wild type mouse tumors (Figure 2). It was also observed that the index of apoptosis is slightly higher in the wild type mouse tumors than in AT_2_-KO mouse tumors, although there was no statistical difference between the two groups (Figure 3). In addition, tumor vessel density was significantly higher in AT_2_-KO mice than in wild type mice (Figure 4). At a glance, the *in vivo *results show that growth of PAN02 cells was significantly faster in the AT_2_-KO environment than in the wild type environment, most likely due to a high degree of cell proliferation. Higher tumor vessel density may also be associated with faster tumor growth in the AT_2_-KO mice.

Following the *in vivo *mouse study, *in vitro *studies were carried out to determine the mechanism by which AT_2 _receptor expression in stromal cells modifies the growth of pancreatic carcinoma cells. In the first *in vitro *experiment, the effect of AT_2 _receptor over-expression in either wild type or AT_2_-KO MSFs was evaluated in co-culture with PAN02 cells. Results clearly indicate that AT_2 _receptor over-expression significantly attenuates growth of co-cultured PAN02 cells. However, this attenuation was completely abolished by the addition of a low concentration of Ang II in the presence of the AT_2 _receptor-specific blocker PD123319 (Figure 5). Since the contribution of MSFs to cell proliferation is approximately one third of the total cell proliferation (MSF + PAN02), since MSF cell proliferation was not influenced by the status of AT_2 _receptor expression (Figure 5) nor by the presence of Ang II or the AT_2 _antagonist (data not shown), and since PAN02 cells do not express Ang II receptors, the growth of PAN02 cells appears to be indirectly regulated by the MSFs. This experiment nicely recapitulates results obtained from the mouse study (Figure 1). Furthermore, VEGF expression in MSFs was shown to be suppressed by Ang II-AT_2 _receptor signaling (Figure 6), implying that AT_2 _receptor expression-dependent growth attenuation may be mediated by the attenuation of VEGF production in stromal fibroblasts. In support of this, the VEGF positive cell numbers were higher in AT_2_-KO mouse tumors than in the wild type mouse tumors (Figure 4). Taken together, these results strongly suggest that AT_2 _receptor signaling in stromal cells plays an important role in inhibition of tumor growth. As this research shows, tumor growth regulation is indirectly controlled through stromal cells. The significance of tumor stromal cells in tumor growth is widely accepted [43] and further emphasized by another recent report [44]. The mechanism by which tumor stromal fibroblasts regulate tumor growth has not been rigorously studied. However, Sugimoto *et al*. suggest that hepatocyte growth factor produced in fibroblasts controls tumor growth[45]. Since Ang II is known to be produced in fibroblasts and acts as a local cell growth regulator [10,15], it is reasonable to speculate that Ang II also plays a role as a local mediator for tumor growth. In support of this speculation, Fujimoto *et al*. [39] have reported AT_1 _receptor over-expression in human pancreatic cancer tissues and AT_1 _receptor-mediated growth regulation in pancreatic cancer cells. Furthermore, Anandanadesan has also reported that Ang II stimulates VEGF expression in a panel of human pancreatic cancer cell lines [38]. The present study also indicates that tumor stromal fibroblasts appear to be a rich source of VEGF (Figure 4).

It is well known that the Ang II receptor has two major isoforms, and their signaling is associated with cell proliferation and apoptosis [20]. The major isoform, the AT_1 _receptor, is expressed in a wide variety of tissues, and its signaling functions in a variety of pathophysiological reactions, including constriction of blood vessels, induction of cell proliferation and expression of proto-oncogenes such as c-fos, c-myc and c-jun [46]. The second major isoform, the AT_2 _receptor, is abundantly expressed in fetal tissues, but its expression declines rapidly after birth [14]. Multiple studies have shown that AT_2 _receptor signaling counteracts the biological effects mediated by AT1 receptor signaling, including inhibition of cell proliferation [41,42,47]. Therefore, the delicate balance between the activities of these two receptors plays an important role in the pathophysiology of various diseases[20]. Accordingly, AT_2 _receptor-deficiency-induced tumor growth stimulation may be mediated at least in part through Ang II-AT_1 _receptor signaling in either stromal cells or cancer cells. Indeed, it has been well documented that Ang II, besides its conventional physiological actions, displays characteristics of a growth factor [48]. The AT_2 _receptor signaling-dependent cell growth attenuation reported here is in good agreement with earlier studies [41,49]. In these studies, growth of vascular endothelial cells and smooth muscle cells were shown to be attenuated by AT_2 _receptor-mediated Ang II signaling. Although these studies did not clarify the potential second messenger that controls cell growth, the present study suggests that AT2 receptor-mediated attenuation of VEGF production is a potential mechanism for AT_2 _receptor expression-dependent growth attenuation of pancreatic carcinoma.

Based on observations by other researchers[20,21,48,50,51] and findings in the present study, it is clear that the AT_2 _receptor plays an important role in tumor growth in rodents. To the best of our knowledge, this is the first report to describe the involvement of AT_2 _receptor mediated signaling in controlling the growth of pancreatic adenocarcinoma at least in part by attenuating stromal fibroblast-dependent VEGF production. However, whether AT_2 _receptor expression indeed plays an important role in human pancreatic cancer growth must be clarified by human clinical studies.

## Conclusion

The present study clearly indicates that the Ang II-AT_2 _receptor signaling plays an important role in the growth regulation of pancreatic adenocarcinoma. Thus, it is suggested that the AT_2 _receptor could be an important target for cancer therapy/chemoprevention.

## Abbreviations

ABC: avidin-biotin peroxidase complex; Ang II: angiotensin II; AT_2_: angiotensin II (Ang II) type 2 receptor; AT_2_-KO: AT_2 _receptor-deficient; VEGF: vascular endothelial growth factor; EGF: epidermal growth factor; FBS: fetal bovine serum; MAPK: mitogen-activated protein kinase; MEK: MAPK kinase 1; MSFs: mouse skin fibroblasts; MTT: Methylthiazol Tetrazolium; PAN02: pancreatic carcinoma; PDAC: pancreatic ductal adenocarcinoma; TUNEL: terminal Deoxynucleotidyltransferase-Mediated dUTP Nick End Labeling; vWF: von Willebrand factor.

## Competing interests

The authors declare that they have no competing interests.

## Authors' contributions

CD, NE and AK equally contributed to this study. CD, NE, AK, NO, DU, RA, LP, YI and MT were responsible for the study design, experimental work, data evaluation and analysis, and drafting the manuscript. DKM, DT, and ST were consulted extensively in the experimental design and interpretation of results, as well as in the preparation of the manuscript. MT was the research supervisor and participated in the study design, assessment of the results, and drafting the manuscript. All authors read and approved the manuscript.

## Pre-publication history

The pre-publication history for this paper can be accessed here:

http://www.biomedcentral.com/1471-2407/10/67/prepub
